# The long noncoding RNA linc-NeD125 controls the expression of medulloblastoma driver genes by microRNA sponge activity

**DOI:** 10.18632/oncotarget.16049

**Published:** 2017-03-09

**Authors:** Pietro Laneve, Agnese Po, Annarita Favia, Ivano Legnini, Vincenzo Alfano, Jessica Rea, Valerio Di Carlo, Valeria Bevilacqua, Evelina Miele, Angela Mastronuzzi, Andrea Carai, Franco Locatelli, Irene Bozzoni, Elisabetta Ferretti, Elisa Caffarelli

**Affiliations:** ^1^ Center for Life NanoScience@Sapienza, Istituto Italiano di Tecnologia, 00161 Rome, Italy; ^2^ Department of Molecular Medicine, Sapienza University of Rome, 00161 Rome, Italy; ^3^ Institute of Molecular Biology and Pathology, National Research Council, 00185 Rome, Italy; ^4^ Department of Biology and Biotechnology, Sapienza University of Rome, 00185 Rome, Italy; ^5^ Department of Hematology/Oncology and Stem Cell Transplantation, Bambino Gesù Children's Hospital, IRCCS, 00165 Rome, Italy; ^6^ Department of Neuroscience and Neurorehabilitation, Neurosurgery Unit, Bambino Gesù Children's Hospital, IRCCS, 00165 Rome, Italy; ^7^ University of Pavia, Corso Strada Nuova, 27100 Pavia, Italy; ^8^ Institute Pasteur Fondazione Cenci-Bolognetti, Sapienza University of Rome, 00185 Rome, Italy; ^9^ Department of Experimental Medicine Sapienza University of Rome, 00161 Rome, Italy; ^10^ Neuromed Institute, 86077 Pozzilli, Italy; ^11^ Present addresses: Center for Genomic Regulation, 08003 Barcelona, Spain; ^12^ Present addresses: Virology Program, INGM-Istituto Nazionale di Genetica Molecolare, 20122 Milan, Italy; ^13^ Present addresses: Department of Hematology/Oncology and Stem Cell Transplantation, Bambino Gesù Children's Hospital, IRCCS, 00165 Rome, Italy

**Keywords:** long noncoding RNAs, competing endogenous RNAs, microRNAs, cancer driver genes, Group 4 medulloblastoma

## Abstract

Long noncoding RNAs (lncRNAs) are major regulators of physiological and disease-related gene expression, particularly in the central nervous system. Dysregulated lncRNA expression has been documented in several human cancers, and their tissue-specificity makes them attractive candidates as diagnostic/prognostic biomarkers and/or therapeutic agents. Here we show that linc-NeD125, which we previously characterized as a neuronal-induced lncRNA, is significantly overexpressed in Group 4 medulloblastomas (G4 MBs), the largest and least well characterized molecular MB subgroup. Mechanistically, linc-NeD125 is able to recruit the miRNA-induced silencing complex (miRISC) and to directly bind the microRNAs miR-19a-3p, miR-19b-3p and miR-106a-5p. Functionally, linc-NeD125 acts as a competing endogenous RNA (ceRNA) that, sequestering the three miRNAs, leads to de-repression of their targets CDK6, MYCN, SNCAIP, and KDM6A, which are major driver genes of G4 MB. Accordingly, linc-NeD125 downregulation reduces G4 cell proliferation. Moreover, we also provide evidence that linc-NeD125 ectopic expression in the aggressive Group 3 MB cells attenuates their proliferation, migration and invasion.

This study unveils the first lncRNA-based ceRNA network in central nervous system tumours and provides a novel molecular circuit underlying the enigmatic Group 4 medulloblastoma.

## INTRODUCTION

Medulloblastoma is the most common malignant paediatric brain tumour [1]. Recent transcriptomics and genomics analyses of large human primary tumour cohorts assigned MBs to four molecularly distinct subgroups, each characterized by specific developmental origins, molecular features, and prognoses [1–4]. The best characterized WNT and SHH subgroups have been causally linked to altered Wingless and Sonic Hedgehog developmental cascades, respectively [1]. Larger gaps remain in our understanding of the signalling pathways underlying Group 3 (G3) and Group 4 (G4) MBs, which account for 60% of all prognoses and present the greatest clinical challenges [4]. G3, the subgroup with the worst outcomes and the highest metastasis rates (75%), is characterized by a MYC-activation signature [1]. G4 tumours have a better prognosis, and are the most common MBs characterized by a neuronal signature with over-representation of genes involved in neuronal differentiation and development [1–4]. Genomic analyses highlighted subgroup-enriched dysregulated genes, the so-called driver genes, altered by single nucleotide variants (SNVs) or somatic copy number aberrations (SCNAs) [1, 2]. However, driver gene expression might also be altered by defects in transcriptional [5] or post-transcriptional regulatory mechanisms, as those involving microRNAs (miRNAs) [6].

Increasing emphasis has been recently placed on the potential roles of long noncoding RNAs (lncRNAs) as gene expression regulators in human nervous system physiology [7]. Aberrant lncRNA expression has been documented in neurodevelopmental, neurodegenerative, and neuro-oncological disorders [8]. In the latter setting, lncRNAs are emerging as critical players: their differential expression in gliomas and neuroblastomas has been actively investigated [9, 10], but very little is known about their roles in MB.

We recently identified a novel human long intergenic noncoding RNA (lincRNA), as the host gene for miR-125b-1. It is specifically induced during *in vitro* differentiation of neuronal tumour cell lines—hence its name: Neuronal Differentiation lncRNA hosting miR-125 (linc-NeD125) [11].

In this study, we explored the roles it plays in brain cancer and discover that linc-NeD125 is an essential node in a novel regulatory network in G4 MB, the most prevalent and pathogenetically enigmatic class of MBs. We demonstrate that, when expressed at the high levels found in G4 MBs, linc-NeD125 functions as a competing endogenous RNA (ceRNA) that, sequestering miR-19a-3p, miR-19b-3p, and mir-106a-5p, de-represses the expression of their targets *CDK6*, *MYCN*, *SNCAIP* and *KDM6A*, major driver genes of G4 MB. Remarkably, we revealed a role for linc-NeD125 in reducing G4 MB cell proliferation and G3 MB cell aggressiveness *in vitro*.

## RESULTS

### Linc-NeD125 is targeted by specific miRNAs

**Figure 1:**
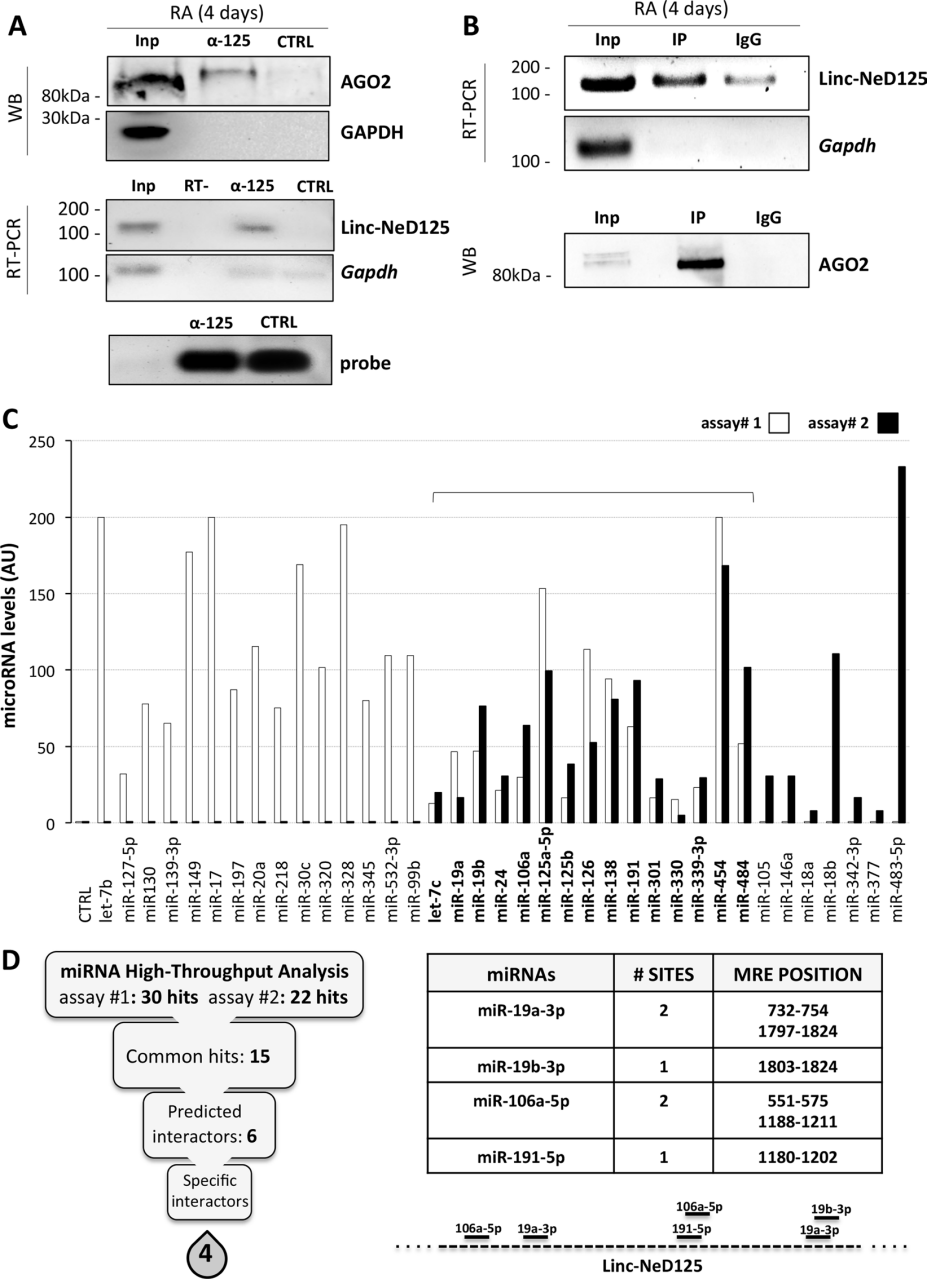
Identification of linc-NeD125 interactors (**A**) Linc-NeD125 RNA pull-down assay in RA-treated BE(2)-C cells. Upper panel: Western blot analysis of AGO2 and GAPDH in Input (Inp), linc-NeD125 probe-bound (α-125) and non specific probe-bound (CTRL) protein fractions. Middle panel: levels of *Linc-NeD125* and *Gapdh* RNAs in Inp, α-125 and CTRL RNA fractions. RT- sample was used as negative control. Lower panel: fractionation on denaturing agarose gel of specific (α-125) and unspecific (CTRL) biotinylated probes. (**B**) AGO2 CLIP assay. Upper panel: RNA analysis from RA-treated BE(2)-C cells of Linc-NeD125 or *Gapdh* as negative control in Input fraction (Inp) and extracts immunoprecipitated with AGO2 (IP) or IgG (IgG). Lower panel: Western blot of AGO2 in AGO2- (IP) or IgG- (IgG) immunoprecipitated cell extracts, or in Input sample (Inp) as control. (**C**) Levels of miRNAs associated with linc-NeD125 in pull-down assays #1 (white bars) and #2 (black bars). Common hits are bolded. Enrichments refer to control samples (CTRL), set as 1. Data are normalized to ath-miR159a levels and expressed as arbitrary units (AU). (**D**) Left panel: scheme summarizing the filtering process identifying specific linc-NeD125 interactors. Right panel: number and positions of miR-19a-3p, miR-19b-3p, miR-106a-5p, miR-191a-5p MREs on linc-NeD125 sequence. Locations of the 6 MREs on linc-NeD125 are schematised below.

In human BE(2)-C neuroblastoma cells, linc-NeD125 is localized in the cytoplasm [11]. This finding, together with preliminary bioinformatics analyses revealing multiple miRNA response elements (MREs) throughout its length (Supplementary Table 1), suggested that linc-NeD125 might act as a ceRNA. To explore this hypothesis, we performed RNA pull-down assays in BE(2)-C cells treated with retinoic acid (RA), which triggers linc-NeD125 expression approximately 6-fold compared to untreated cells [11]. These assays showed that linc-NeD125 was associated with Argonaute 2 (AGO2), a major component of the miRNA-induced silencing complex (miRISC) where miRNAs interact with their mRNA targets (Figure 1A). Linc-NeD125-AGO2 interaction was confirmed by crosslinking immunoprecipitation (CLIP) experiments (Figure 1B). The two approaches demonstrate that linc-NeD125 is able to recruit the miRISC, a pre-requisite to function as a ceRNA.

To identify the miRNAs possibly associated with linc-NeD125 in the miRISC, high-throughput qRT-PCR analysis was performed on complexes precipitated from two distinct linc-NeD125 pull-down assays. 15 miRNAs were found in both experiments (Figure 1C), 6 of which were predicted to target linc-NeD125 according to the miRanda algorithm (Figure 1D, left panel, and Supplementary Table 1). The same tool was used to eliminate 2 of the 6 miRNAs that could bind the pull-down bait, leaving a short list of 4 miRNAs—namely miR-19a-3p, miR-19b-3p, miR-106a-5p and miR-191-5p—which are specifically bound by linc-NeD125 (Figure 1D, right panel).

### Linc-NeD125 is expressed in MBs and upregulated in G4 subgroup

**Figure 2:**
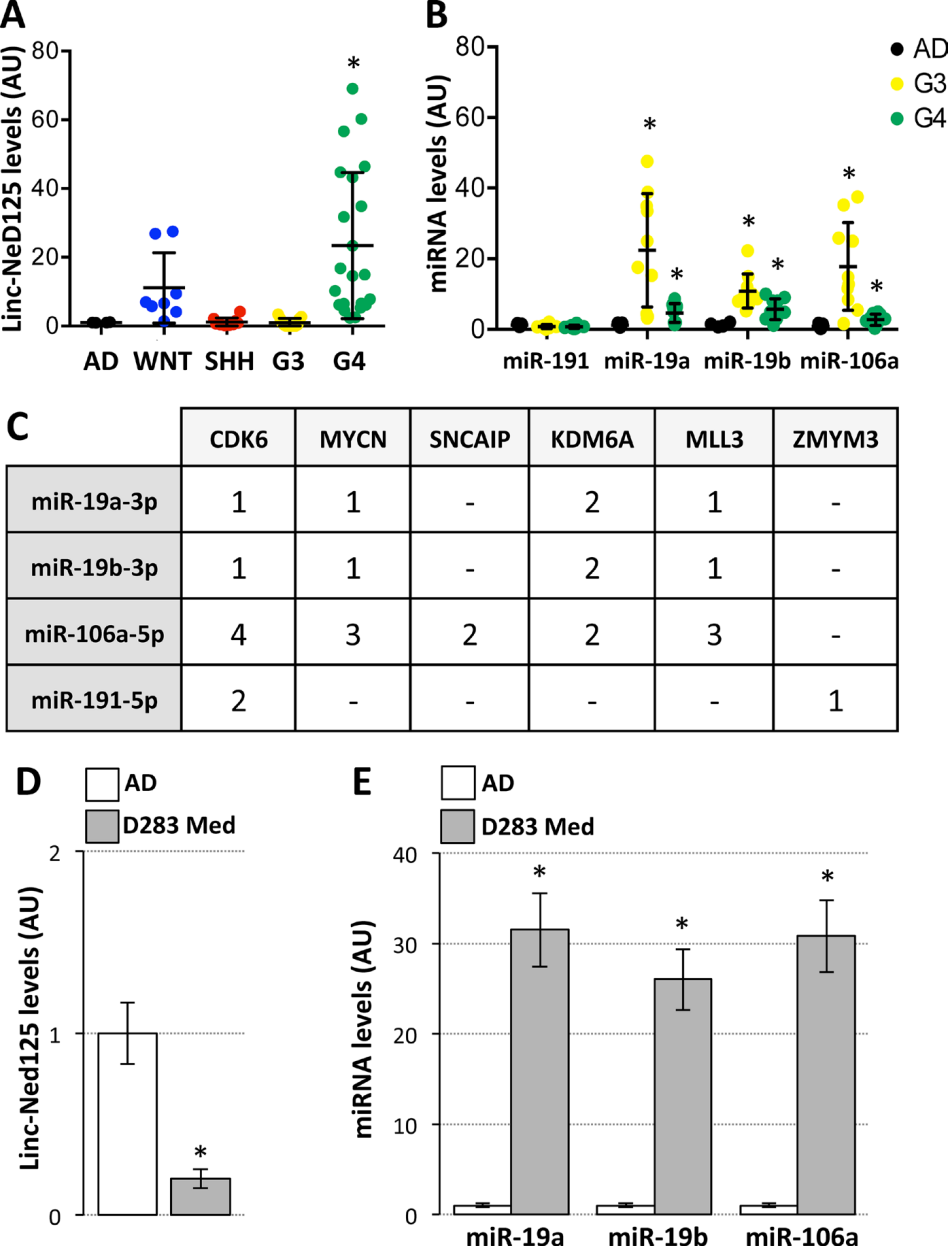
Expression of linc-NeD125 and interacting miRNAs in primary MBs and D283 Med cells (**A**) Linc-NeD125 expression in 51 primary MBs (coloured dots; subgroup distribution: WNT = 8; SHH = 10; G3 = 13; G4 = 20) and 10 normal cerebella (AD, black dots). Results (means+/−s.d.) expressed in arbitrary units (AU) are normalized to the mean value of 4 housekeeping genes (*p < 0.05). (**B**) MiR-191a-5p, miR-19a-3p, miR-19b-3p and miR-106a-5p expression in G3 (yellow dots) or G4 MBs (green dots) *vs* normal cerebella (AD, black dots). Results (means+/−s.d.) expressed in arbitrary units (AU) are normalized to levels of U6 snRNA (*p < 0.05). (**C**) Predicted miRNA target sites within the 3′UTR of G4 MB driver genes. (**D**) Linc-NeD125 is under-expressed in D283 Med cells (gray bar) compared to normal cerebella (AD, white bar). Data analysis as in (A). (**E**) Up-regulation of miR-19a-3p, miR-19b-3p and miR-106a-5p in D283 Med cells (gray bars) compared to normal cerebella (white bars, AD). Results expressed as in (B).

The experiments in tumour-derived neuronal cells provided evidence that linc-NeD125 is a potential ceRNA. Given the increasing evidence for the involvement of lncRNAs as ceRNAs in neuronal cancer-associated networks [12], we asked whether linc-NeD125 may play this role in MBs. Taking advantage of a large number of available human specimens, we evaluated linc-NeD125 expression in a cohort of 51 primary tumours (Supplementary Table 2), representing all four MB subgroups in proportions reflecting their incidence in the population [1]. As shown in Figure 2A, linc-NeD125 was expressed in all subgroups and significantly upregulated (20-fold increase on average) in G4 MB, compared to normal cerebellum. Levels found in G4 tumors were approximately twice as high as those in WNT MBs and roughly 20 times those of the SHH and G3 tumours.

### miR-19a-3p, miR-19b-3p and miR-106a-5p repress G4 MB driver gene expression

To determine linc-NeD125 contribution to G4 MB, we checked whether genes relevant for this tumour subgroup were specific targets for the bound miRNAs. We initially assessed miRNA occurrence in G4 primary tumors. As shown in Figure 2B, only miR-19a-3p, miR-19b-3p, and miR-106a-5p were significantly overexpressed in tumour specimens, with 2- to 4-fold increases over control levels.

Subsequent *in silico* analysis showed that the G4 driver genes, namely the proto-oncogenes CDK6 and MYCN, the α-synuclein-interacting protein SNCAIP, the histone H3 lys27 demethylase KDM6A, the H3K4 methyltransferase MLL3 and the zinc finger protein ZMYM3 [1], share a significant number of predicted targeting microRNAs (Supplementary Table 3), suggesting a coordinated regulation. Among those, we found that the microRNAs bound by linc-NeD125, miR-19a-3p, miR-19b-3p and miR-106a-5p, were predicted to pleiotropically repress five G4 driver genes (Figure 2C).

To validate this prediction we used, as an *in vitro* model system, the D283 Med cells. They are G3-derived MB cells [13] that show low levels of linc-NeD125 (Figure 2D) allowing us to upregulate its expression as observed in G4 tumours.

**Figure 3:**
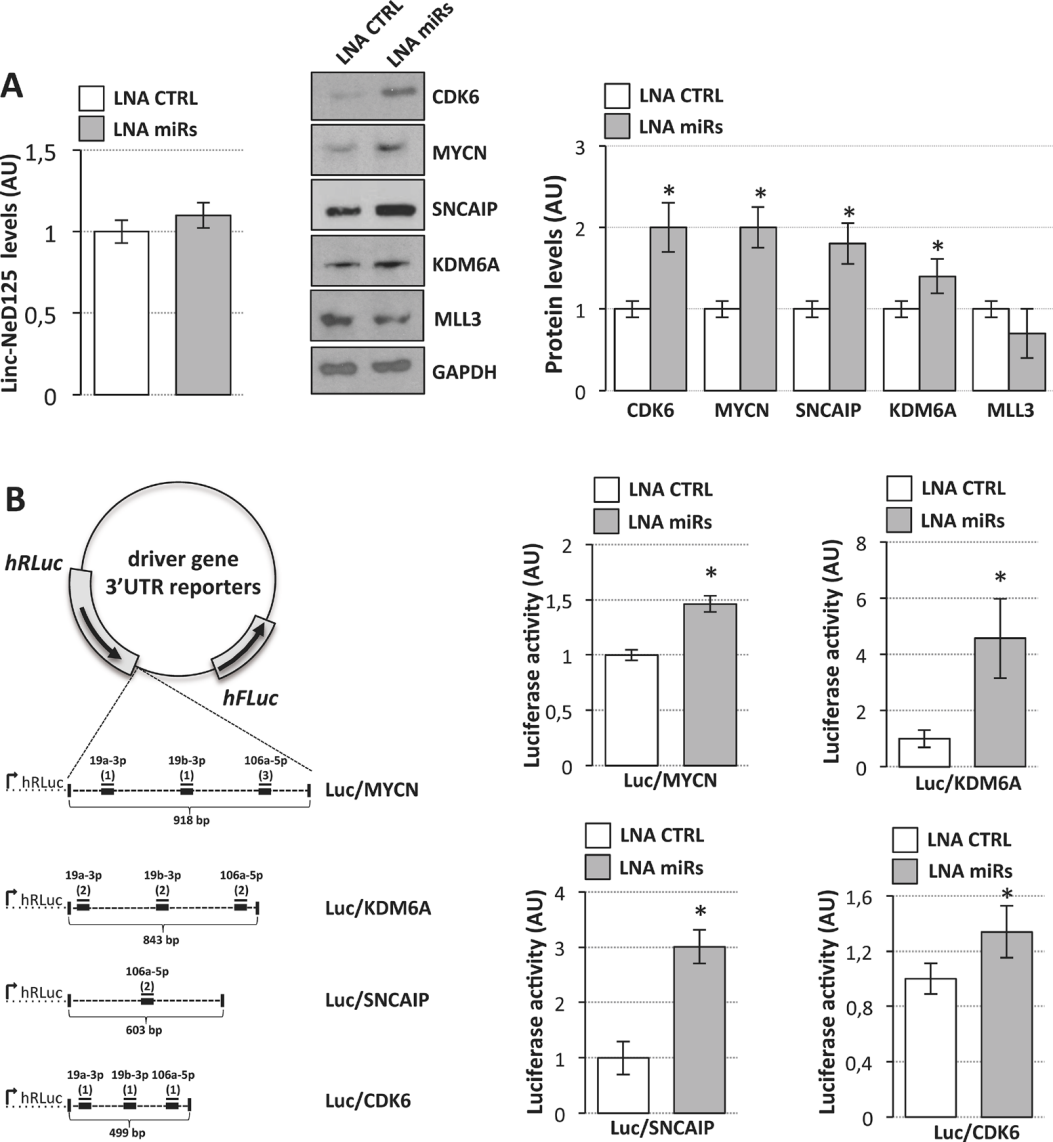
Interaction of miR-19a-3p, miR-19b-3p and miR-106a-5p with G4 driver genes (**A**) miRNAs regulate driver gene expression. Left panel: linc-NeD125 expression in D283 Med cells transfected with LNA inhibitors targeting miR-19a, miR-19b and miR-106a (LNA miRs, gray bar) or with scrambled LNA (LNA CTRL, white bar). Middle panel: Western blot analysis of five G4 MB driver gene protein products in D283 Med cells transfected with LNAs against miR-19a, miR-19b, miR-106a (LNA miRs) or with scrambled LNA (LNA CTRL). GAPDH: loading control. Right panel: protein level densitometric analysis. Results (means+/−s.d.) from three biological replicates (*p < 0.05) are expressed in arbitrary units (AU). (**B**) Luciferase assays in D283 Med cells. Left panel: representation of luciferase/driver gene 3′UTR reporter constructs. MREs are indicated as thick lines, miRNAs as thin lines. For each miRNA, number of MREs are reported in brackets. Right panels: activity of Renilla luciferase expressed from the constructs shown in the left panel, in the presence of specific LNAs (LNA miRs, gray bars) or scrambled LNA (LNA CTRL, white bars). Renilla luciferase activity (means+/−s.d. from three biological replicates) expressed as arbitrary units (AU), is normalized over firefly luciferase activity (internal control) and referred to CTRL sample, set as 1.

Given the high expression levels of miR-19a-3p, miR-19b-3p and miR-106a-5p in D283 Med cells (Figure 2E), miRNA loss-of-function experiments were performed. Co-transfection of Locked Nucleic Acids (LNAs) against the three miRNAs did not alter linc-NeD125b levels (Figure 3A, left panel), while caused an increase of CDK6, MYCN, SNCAIP and KDM6A protein levels (ranging from ~1.4 to ~2-fold over LNA-control treated cells) (Figure 3A, middle and right panels), demonstrating that the corresponding genes are targets of the three miRNAs bound by linc-NeD125.

Further demonstration that the three miRNAs together are able to specifically interact with *CDK6*, *MYCN*, *SNCAIP* and *KDM6A* mRNAs derived from luciferase reporter assays. As schematized in Figure 3B (left panel), portions of their 3′UTRs were cloned downstream of the r-luc ORF and expressed in D283 Med cells together with scramble or miRNA targeting LNAs. Histograms in Figure 3B (right panels) show an increase of luciferase activity indicating the specificity of the three miRNA interaction.

### Linc-Ned125 de-represses G4 MB driver gene expression by sequestering miR-19a-3p, miR-19b-3p, and miR-106a-5p

To explore linc-NeD125 involvement in the control of G4 MB driver genes and the possibility that it is mediated by its miRNA sponge activity, we designed constructs expressing mature wild type linc-NeD125 (Linc-125) or a mutant derivative defective in miRNA binding activity (mLinc-125) (Supplementary Figure 1). The latter was planned by introducing single point mutations in MRE sites corresponding to miRNA seed positions 4 and 5 [14]. Importantly, we ensured that none of the mutations created new miRNA-binding sites.

**Figure 4:**
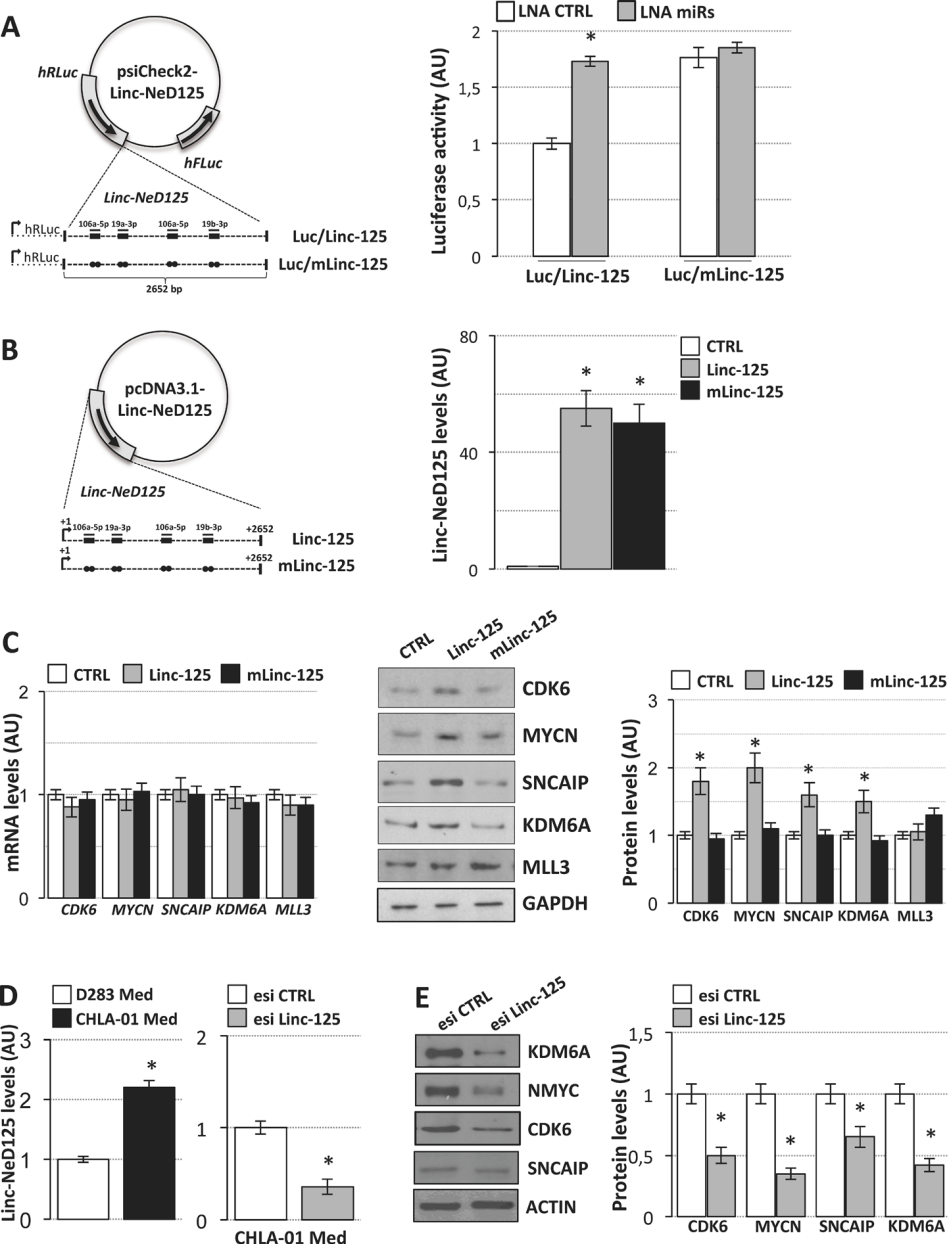
Linc-NeD125 overexpression and downregulation in MB cells (**A**) Luciferase assays in D283 Med cells. Left panel: representation of wild type (Luc/Linc-125) or mutant (Luc/mLinc-125) luciferase/linc-NeD125 reporter constructs. Wild type MREs are indicated as thick lines, miRNAs as thin lines, point mutations as dots (See Supplementary Figure 1 for mutant sequences). Right panel: activity of Renilla luciferase expressed from wild type (bars Luc/Linc-125) or mutant construct (bars Luc/mLinc-125) in the presence of LNA inhibitors (LNA miRs, gray bars) or scrambled control LNA (LNA CTRL, white bars). Renilla luciferase activity (means+/−s.d. from three biological replicates), expressed as arbitrary units (AU), is normalized over firefly luciferase activity (internal control) and referred to wild type CTRL sample, set as 1. (**B**) Linc-NeD125 overexpression in D283 Med cells. Left panel: constructs are represented as in (A). Right panel: levels of linc-NeD125 in D283 Med cells transfected with Linc-125 (gray bar), mLinc-125 (black bar) or with the empty vector (CTRL, white bar). Results, expressed as arbitrary units (AU) (means+/−s.d.) are normalized to the mean value of 4 housekeeping genes (*p < 0.05) and referred to CTRL sample, set as 1. (**C**) Left panel: expression levels of *CDK6, MYCN, SNCAIP, KDM6A* and *MLL3* mRNAs in D283 Med transfected with constructs expressing wild type (Linc-125) or mutant linc-NeD125 (mLinc-125). Middle panel: Western blot of CDK6, MYCN, SNCAIP, KDM6A and MLL3 proteins in D283 Med cells transfected as described in left panel. GAPDH: loading control. MLL3 analysis was included as a negative control. Right panel: protein level densitometric analysis. Results are expressed as means+/−s.d. from at least three biological replicates (*p < 0.05) and referred to CTRL samples set as 1. MiRNA levels evaluated in the same conditions are reported in Supplementary Figure 2. (**D**) Left histogram: comparative qRT-PCR analysis of Linc-NeD125 in D283 Med (white bar) and CHLA-01 Med cells (black bar). Right histogram: levels of linc-NeD125 in CHLA-01 Med cells transfected with Linc-NeD125 esiRNA (esi Linc-125, gray bar) or scrambled esiRNA (esi CTRL, white bar). Data analysis as in (C). (**E**) Left panel: Western blot of CDK6, MYCN, SNCAIP, and KDM6A proteins upon transfection of Linc-NeD125 esiRNA (esi Linc-125, gray bars) or scrambled esiRNA (esi CTRL, white bars) in CHLA-01 cells. ACTIN: loading control. Right panel: protein level densitometric analysis. Data analysis as in (C).

To verify the specificity of miRNA-linc-NeD125 interaction, we cloned the wild type and mutant linc-NeD125 into luciferase reporter vectors (Figure 4A, left panel) and transfected them into D283 Med cells, along with the LNAs complementary to miR-19a-3p, miR-19b-3p, and miR-106a-5p. As shown in Figure 4A (right panel), cells transfected with the LNAs and wild type linc-NeD125 (Luc/Linc-125) exhibited increased luciferase activity (1.5-fold over controls), but no change was observed in cells transfected with the mutant transcript (Luc/mLinc-125). These results confirm the specificity of the miRNA/linc-NeD125 interaction and the inability of the mutant transcript to bind the three miRNAs.

**Figure 5:**
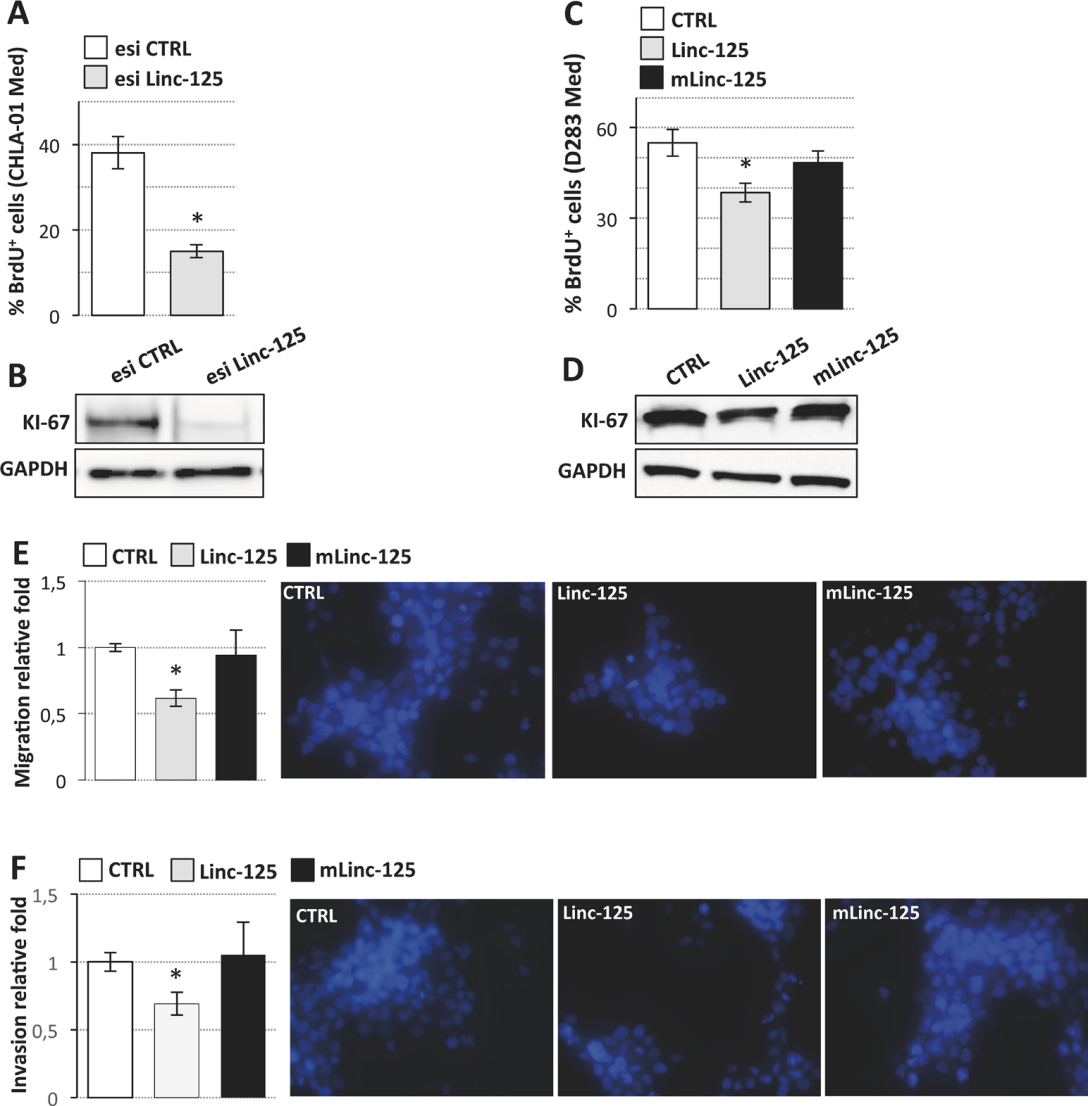
Effects of linc-NeD125 overexpression or knockdown on MB cell properties (**A**) BrdU assay of CHLA-01 Med cells transfected with Linc-NeD125 esiRNA (esi Linc-125, gray bar) or scrambled esiRNA (esi CTRL, white bar). Results (means+/−s.d.) are expressed in percentage of BrdU positive cells from three biological replicates (*p < 0.05). (**B**) Western blot analysis of KI-67 from cells treated as in (A). GAPDH: loading control. (**C**) BrdU assay of D283 Med cells transfected with Linc-125 (gray bar), mLinc-125 (black bar) or empty vector (CTRL, white bar). Details as in (A). (**D**) Western blot analysis of KI-67 from cells treated as in (C). GAPDH: loading control. (**E**) Cell migration. D283 Med cells transfected as in (C) were Hoechst-stained and counted after a 24 h-migration. Histogram represents the number of migrating cells from three biological replicates, relative to CTRL set as 1. Pictures of representative fields are reported aside. (**F**) Cells invasion. Details as in (E).

For functional analyses, the wild type and mutant linc-NeD125 sequences were cloned into an expression vector (Figure 4B, left panel) and transfected in D283 Med cells. As shown in Figure 4B (right panel) the two transcripts were overexpressed at levels comparable to those characterizing primary G4 MBs. Their ectopic expression had no effect on the levels of miR-19a-3p, miR-19b-3p, or miR-106a-5p (Supplementary Figure 2), indicating that linc-NeD125 does not regulate their abundance [15]. G4 driver gene mRNA levels were also unaltered (Figure 4C, left panel), suggesting that their regulation by the three miRNAs occurs mainly at the translational level (See Figure 3A, middle panel). In contrast, driver gene protein products were significantly increased by ectopic expression of wild type linc-NeD125 (1.5- to 2-fold increases), as compared with untreated controls (Figure 4C, middle and right panels). No any effect was produced by overexpression of the mutant linc-NeD125. Complementary loss-of-function experiments were carried out in a recently reported G4 *in vitro* model system, the CHLA-01-MED cell line [16], where linc-NeD125 is expressed at higher levels compared to D283 Med cells (Figure 4D, left panel). We found that knockdown of the endogenous linc-NeD125 in CHLA-01-MED cells (Figure 4D, right panel) caused a significant decrease of the four driver gene protein products (Figure 4E), and that this is accompanied by a significant reduction of cell proliferation (Figure 5A, upper panel) and of the proliferation marker KI-67 (Figure 5A, lower panel).

Collectively, these results demonstrate that linc-NeD125 controls the *in vitro* expression of four genes known to drive G4 MB, i.e. *CDK6*, *MYCN*, *SNCAIP*, and *KDM6A*, by competing with their transcripts for binding to miR-19a-3p, miR-19b-3p and miR-106a-5p.

### Linc-NeD125 impairs *in vitro* cell proliferation, migration and invasion of G3 MB cells

We asked whether the observed linc-NeD125-mediated derepression of specific G4 MB driver genes in a G3 MB genetic background led to any phenotypic consequence.

**Figure 6:**
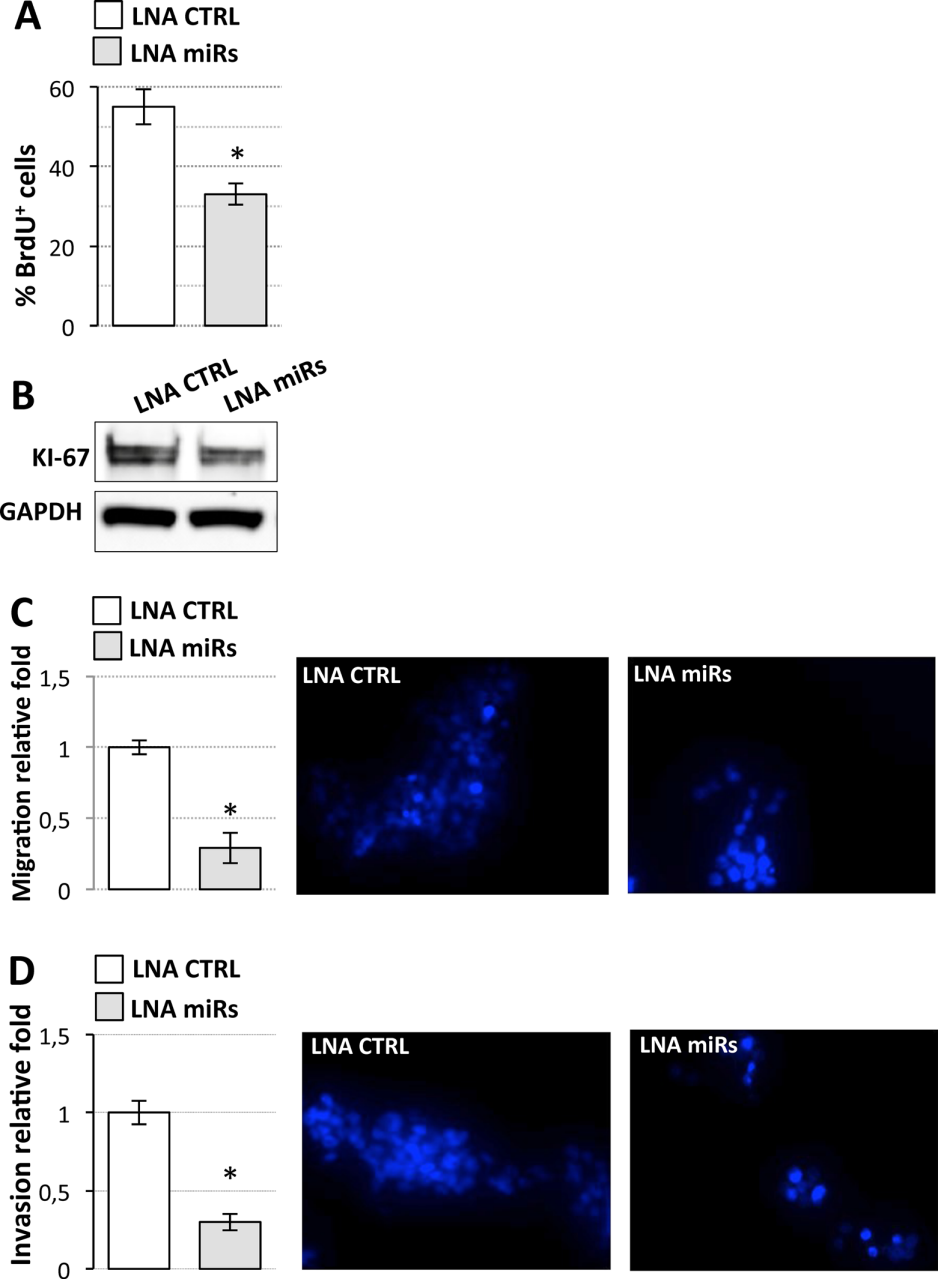
Effects of miR-19a-3p, miR-19b-3p, miR-106a-5p inhibition on D283 Med cell properties (**A**) BrdU assay of D283 Med cells transfected with LNAs against miR-19a-3p, miR-19b-3p, miR-106a-5p (LNA miRs, gray bar) or with a scrambled LNA (LNA CTRL, white bar). Results (means+/−s.d.) are expressed in percentage of BrdU positive cells of three biological replicates (*p < 0.05). (**B**) Western blot analysis of KI-67 from cells treated as in (A). GAPDH: loading control. (**C**) Cell migration. D283 Med cells transfected as in (A) were Hoechst-stained and counted after a 24 h-migration. Histogram represents the number of migrating cells from three biological replicates, relative to CTRL set as 1. Pictures of representative fields are reported aside. (**D**) Cells invasion. Details as in (C).

We assessed G3-derived D283 Med cells’ capacities for proliferation, migration, and invasion after overexpression of wild type or mutant linc-NeD125 (Figure 5B), at the high levels observed in primary G4 tumors. Unexpectedly, the results of BrdU labelling assay, which measures DNA replication rates (Figure 5C), and the analysis of the proliferation marker KI-67 (Figure 5D) showed that cell proliferation was significantly reduced by wild type linc-NeD125 (a decrease of approximately 30%) but was unaffected by the mutant. Migration (Figure 5E) and invasion (Figure 5F) were also reduced (by approximately 40%) in linc-NeD125 overexpressing cells, but no effects were seen in cells expressing the mutant transcript. To confirm that linc-NeD125 biological function depends on its ability to inhibit miRNA activity, we analyzed D283 Med cell proliferation (Figure 6A and 6B), migration (Figure 6C) and invasion (Figure 6D) upon LNA-mediated miRNA sequestration. A decrease of about 40% of cell proliferation and a 70% decrease of migration and invasion of D283 Med cells were observed.

**Figure 7:**
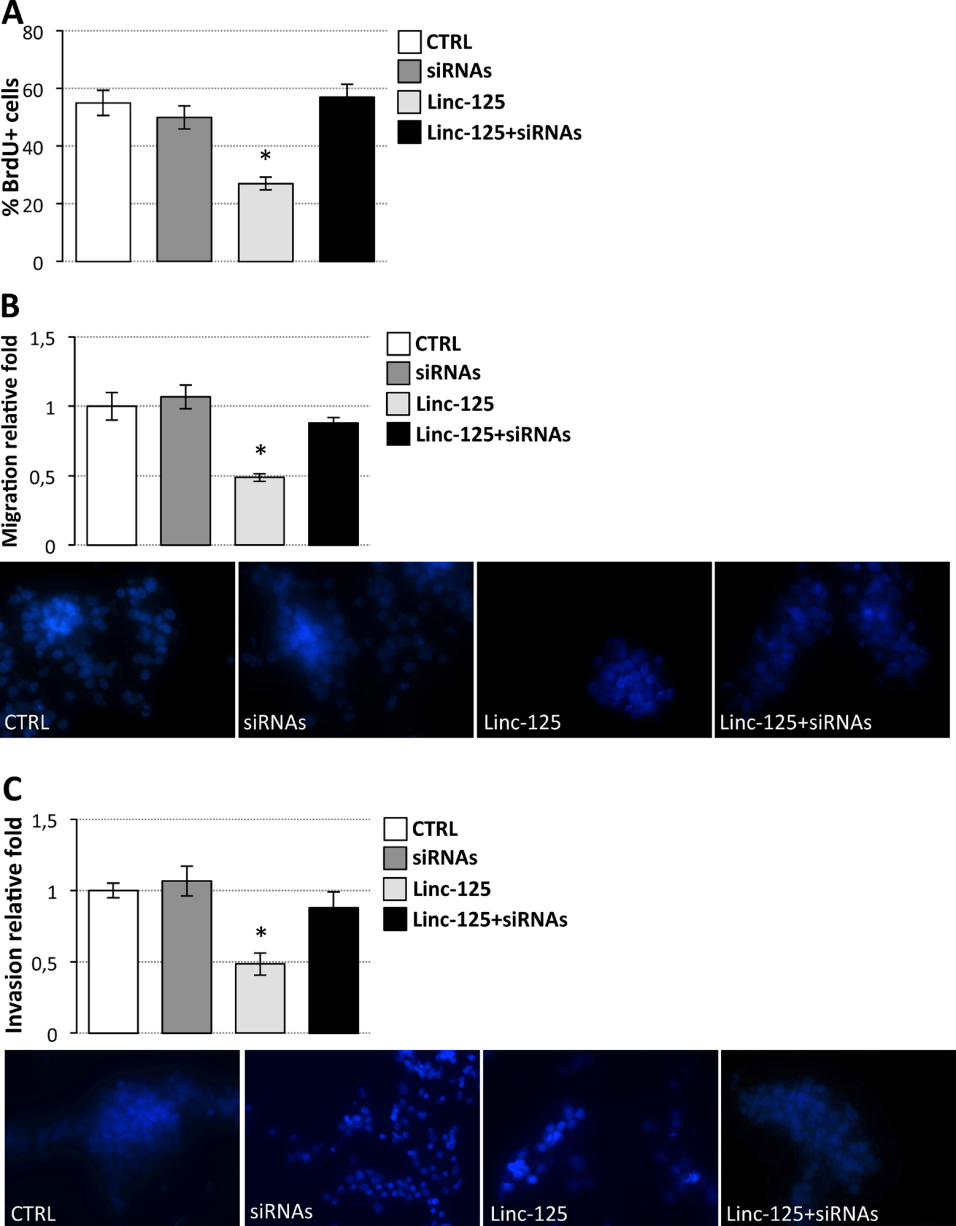
Effects of driver gene downregulation and linc-NeD125 overexpression on D283 Med cell properties (**A**) BrdU assay of D283 Med cells transfected with siRNAs against *CDK6*, *MYCN*, *SNCAIP*, and *KDM6A* (dark gray bar) or with the plasmid overexpressing Linc-NeD125 (Linc-125, light gray bar) or with siRNAs and Linc-125 together (black bar), compared to cells transfected with scrambled siRNAs and empty vector (CTRL, white bar). Results (means+/−s.d.) are expressed in percentage of BrdU positive cells of three biological replicates (*p < 0.05). (**B**) Cell migration. D283 Med cells transfected as in (A) were Hoechst-stained and counted after a 24 h-migration. Histogram represents the number of migrating cells from three biological replicates, relative to CTRL set as 1. Pictures of representative fields are reported below. (**C**) Cells invasion. Details as in (B).

Furthermore, to demonstrate that linc-NeD125 action is directed towards the G4 driver genes CDK6, MYCN, SNCAIP, and KDM6A, linc-NeD125 was ectopically expressed in D283 Med cells while repressing their expression. Figure 7 shows that siRNA-mediated silencing of the driver genes prevents linc-NeD125 from affecting cell proliferation (Figure 7A), migration (Figure 7B) and invasion (Figure 7C).

These results demonstrate that linc-NeD125 ectopic expression in D283 Med cells can effectively attenuate the G3 MB cell capacities for proliferation, migration, and invasiveness, and that these effects are mediated by linc-NeD125 sponge activity.

## DISCUSSION

Roughly 60% of MBs belong to G3 and G4. Both are associated with relatively unfavourable outcomes (particularly G3), and targeted therapies are at their infancy. The pathogenesis of G3 and G4 is still poorly defined: their development cannot be adequately explained by SCNAs and SNVs alone, and it could depend on additional mechanisms operating widely at the epigenetic and/or post-transcriptional levels.

**Figure 8:**
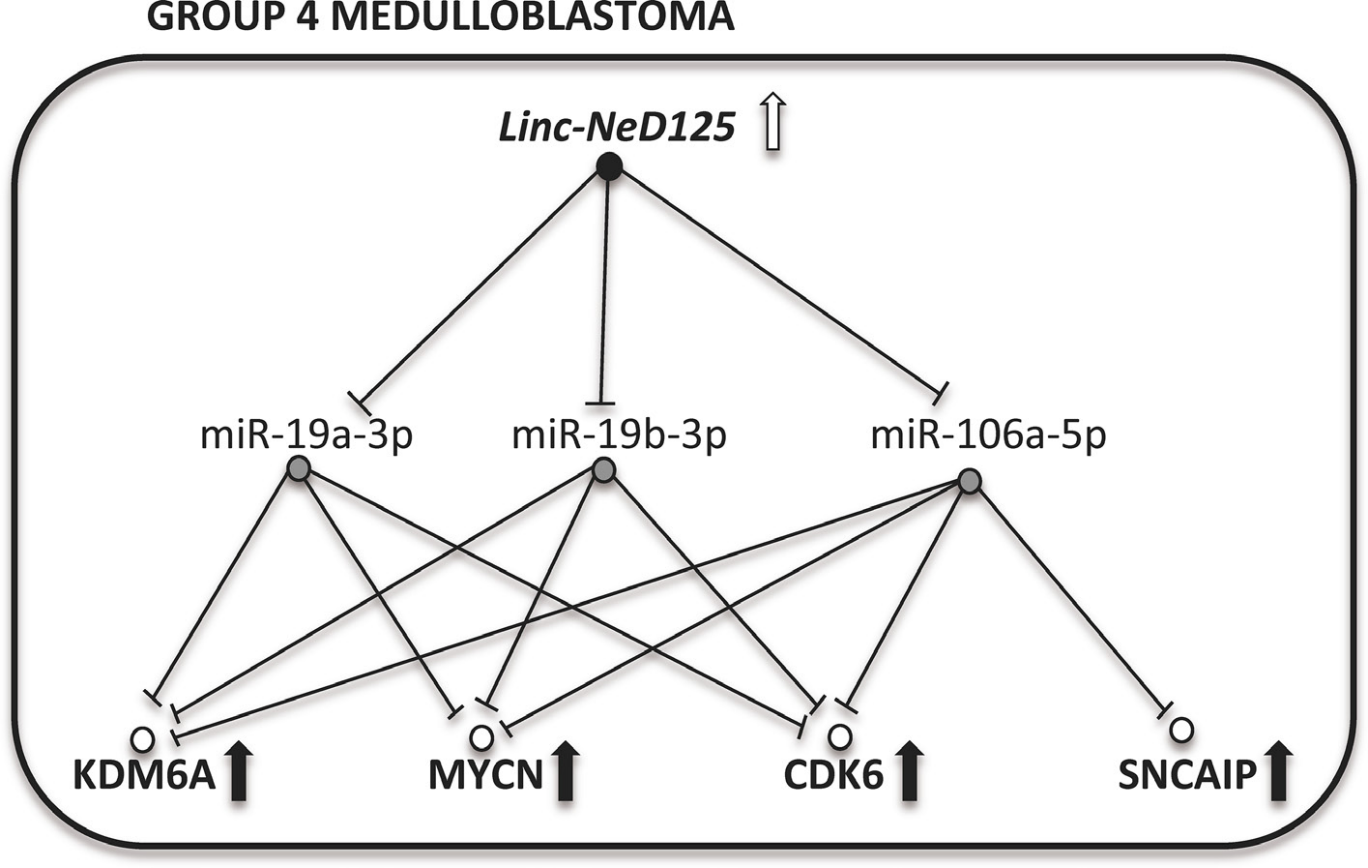
A model of linc-NeD125-dependent ceRNA network in G4 MB Upregulation of linc-NeD125 (open arrow) represses miRNA activity (blocked lines) causing driver gene derepression (black arrows).

Our findings delineate a novel ceRNA network headed by linc-NeD125 (Figure 8), which significantly contributes to the dysregulation of four critical G4 MB driver genes. Linc-NeD125 functions as a natural miRNA sponge, competitively binding and sequestering three endogenous miRNAs—miR-19a-3p, miR-19b-3p and miR-106a-5p—whose targets include *CDK6*, *MYCN*, *SNCAIP*, and *KDM6A* transcripts (Figure 8). This is the first report of a lncRNA-based ceRNA network associated with neuronal cancer [12], and a new example of a single RNA sponge simultaneously controlling a large number of co-regulated targets in cancer [17, 18].

CeRNA activity has been described in different experimental systems and attributed to several RNA species, including lncRNAs, pseudogenes, circular RNAs, and mRNAs [19]. Different models predict the requirement of near-equimolarity among ceRNA network components for regulation to occur [20]. However, natural regulatory networks are likely to be more complex owing to physiological and pathological variations (transient enrichment, local concentration) in RNA levels. Our data indicate that linc-NeD125 behaves as a miRNA sponge in G4 MBs, where its expression is approximately 20-fold higher than that found in normal cerebellar tissues. This conclusion derives from gain-of-function experiments in D283 Med cells, which exhibit the molecular features of primary G3 MB [13], including linc-NeD125 expression levels lower than those found in G4 tumors. This makes them a highly suitable system for mechanistic studies of linc-NeD125 function. Remarkably, linc-NeD125 overexpression in D283 Med cells at levels comparable to G4 tumors elicited the de-repression of multiple G4 MB driver genes, targets of the sponged miRNAs. Linc-NeD125 sponge activity is confirmed by the evidence that overexpression of its mutant derivative, carrying microRNA target sites disrupted at specific positions in the seed, fails to alter G4 MB driver gene expression. Unlike commonly used deletion mutants, this designed site-specific mutant created no novel miRNA binding sites and minimized changes in linc-NeD125 sequence and structure, two features crucial for noncoding RNA activity. Notably, the complementary linc-NeD125 loss-of-function experiment carried out in the G4 MB cell line CHLA-01-MED confirmed its role as a ceRNA regulating G4 driver genes. This, together with the decrease of G4 cell proliferation upon linc-NeD125 knockdown, indicates it may function as an oncogene in G4 MB tumours. *In vivo* studies are required to address this point.

The phenotypic upshot of linc-NeD125 overexpression in a G3 cell model is the acquisition of specific G4 molecular features, namely an increase of the G4 driver gene protein products. Unexpectedly, this was accompanied by a significant attenuation of the high capacity for proliferation, migration and invasion that characterises the high metastatic G3 cells, making them G4-like cells for these capacities. Although the underlying mechanism, dependent on linc-NeD125 ceRNA function, is still unknown, we speculate that it might be related to the neuronal signature that specifically marks G4 MB [1] and that is possibly responsible for the less aggressiveness of this tumour subgroup. In fact, while group 3 has the worst prognosis with less than 50% 5-year survival, group 4 has an about 75% 5-year overall survival [1]. In light of its ability to attenuate the *in vitro* aggressiveness of the metastatic G3 MB cell line and the proliferation of G4 MB cells, linc-NeD125 mode of action merits deeper study for possible therapeutic applications.

In conclusion, post-transcriptional miRNA-mediated crosstalk between linc-NeD125 and protein-coding RNAs appears to play a significant role in G4 MB tumorigenesis. Linc-NeD125 may be a novel driver gene operating upstream of the already identified G4 protein-coding driver genes, and/or it might keep high the levels of driver gene products participating in the maintenance of cancer cell identity.

The results of our study highlight linc-NeD125 as a key player in G4 MB driver gene network and as a novel member of the small but growing list of lncRNAs implicated in human cancerogenesis via the ceRNA mechanism.

## MATERIALS AND METHODS

### Human tissue samples

Surgical specimens of 51 primary MBs were collected at the Bambino Gesù Children's Hospital in Rome (2011–2015). Clinical features and sample treatments are detailed in Supplementary Table 2 and Supplementary Materials and Methods.

### MB molecular subgrouping

Molecular characterization of the 51 tumours was performed as previously described [21]. Details of molecular characterization are shown in Supplementary Table 4.

### Cell lines

BE(2)-C, D283 Med and CHLA-01 Med cells were cultured according to the recommended conditions (ATCC). Details are in Supplementary Materials and Methods.

### Plasmids

pcDNA3.1-Linc-NeD125: the wild-type version was PCR-amplified from cDNA generated from 4 day RA-treated BE(2)-C cells with the oligolucleotides UpBam and DownNot1, and inserted between the BamHI and NotI restriction sites of the pcDNA3.1+ vector. The mutant version was derived by the QuikChange II Site-Directed Mutagenesis Kit (Agilent), using the oligonucleotides Mut1-Mut4. psiCheck2-Linc-Ned125: wild type version was PCR-amplified from pcDNA3.1-Linc-NeD125 with the oligonucleotides UpXho and DownNot1. The mutant was derived from the wild type, as described above. pEZX-MYCN and pEZX-SNCAIP 3′UTR luciferase reporter constructs were purchased from GeneCopoeia. psiCheck2-KDM6A and psiCheck2-CDK6: KDM6A and CDK6 3′UTRs were PCR amplified from genomic DNA with the oligonucleotides UpXho2 and DownNot2 or UpXho3 and DownNot3, respectively. Oligonucleotides are listed in Supplementary Table 5.

### Overexpression and knockdown experiments in D283 Med and CHLA-01 Med cells

Plasmids were transfected (or co-transfected) with Lipofectamine 2000 (Thermo Scientific). LNAs targeting miRNAs (Exiqon) and siRNAs targeting G4 driver genes (Qiagen) were transfected with Hyperfect reagent (Qiagen) at a concentration of 20 nM (each molecule). EsiRNAs (SIGMA Aldrich) were used as in [11] for linc-NeD125 knockdown.

### Expression analyses

Purified RNA was retrotranscribed and analyzed on a ViiA™7 Real-Time PCR System (Applied Biosystems-Thermo Scientific) using best-coverage TaqMan gene expression assays specific for each mRNA. MiRNA expression levels were measured with miRNA-specific Taqman assays (Thermo Scientific) and results were normalized *vs* the endogenous control (snRNA U6). Linc-NeD125 RNA detection was performed using the ViiA™7 Real-Time PCR System with primers 125 FW and 125 REV and normalized to *Gapdh* levels.

### Western blot and antibodies

Western blots were performed as previously described [22], using the antibodies reported in Supplementary Materials and Methods.

### RNA pull-down assay

Linc-NeD125 pull-down assays were performed with biotinylated antisense specific or unspecific probes on extracts from RA-treated BE(2)-C cells, as described [11]. Proteins and RNA were extracted from eluted samples and analyzed by Western blot, RT-PCR or qRT-PCR.

### Crosslinking immunoprecipitation (CLIP) assay

Extracts from RA-treated BE(2)-C cells were immunoprecipitated with 5 μg of AGO2 antibody (Ascenion, 11A9), as described [11]. After extraction using the miRNeasy Mini Kit (Qiagen), immunoprecipitated RNA was retrotranscribed and analyzed by RT-PCR using specific primers for linc-NeD125 or *Gapdh* mRNAs. Protein levels were analyzed by Western blot.

### High-throughput miRNA analysis

MiRNA enrichment was profiled by Applied Biosystems TaqMan^®^ microRNA Arrays A and B (Applied Biosystems), according to the manufacturer's instructions. Details in Supplementary Materials and Methods.

### Luciferase assays

D283 Med cells were co-transfected with constructs expressing Luc/Linc-125, Luc/mLinc-125, Luc/MYCN, Luc/SNCAIP, Luc/CDK6 or Luc/KDM6A transcripts (plasmid: 0.05 ng/ml of transfection mix) and LNAs (20 nM/each, specific or scrambled). 48–72 hours after transfections, cells were lysed and luciferase activity was measured in Glomax Multi+ Detection System (Promega), using Dual-Luciferase Reporter Assay System (Promega).

### Cell proliferation, migration and invasion assays

Cell proliferation was evaluated by Bromodeoxyuridine (BrdU) assay. Migration and invasion assays were performed in Boyden chambers with non-coated or Matrigel-coated membranes, respectively. Cells on the lower surface of the membranes were fixed, Hoechst-stained and counted under microscope. Details in Supplementary Materials and Methods.

### Statistical analysis

Results are expressed as means+/−s.d. from an appropriate number of experiments indicated in Figure Legends. Statistical differences were analyzed by the Mann–Whitney *U*-test for non-parametric values using the GraphPad_Prism software. An adjusted *p*-value < 0.05 was considered as statistically significant.

### Bioinformatics analysis

miRNA/target interactions were predicted with miRanda 3.3 and microRNA.org database. Linc-NeD125 mutant derivative was designed according to miRanda 3.3. Details in Supplementary Materials and Methods.

## SUPPLEMENTARY MATERIALS FIGURES AND TABLES






