# Supplementary material for: The long noncoding RNA linc-NeD125 controls the expression of medulloblastoma driver genes by microRNA sponge activity
Source: Oncotarget. 2017 Mar 9;8(19):31003–15. doi: 10.18632/oncotarget.16049 (PMC5458184; doi:10.18632/oncotarget.16049)
Supplement: Supplementary file 1 [file oncotarget-08-31003-s001.pdf]

The long noncoding RNA linc-NeD125 controls the expression of medulloblastoma driver genes by microRNA sponge activity

Supplementary Materials

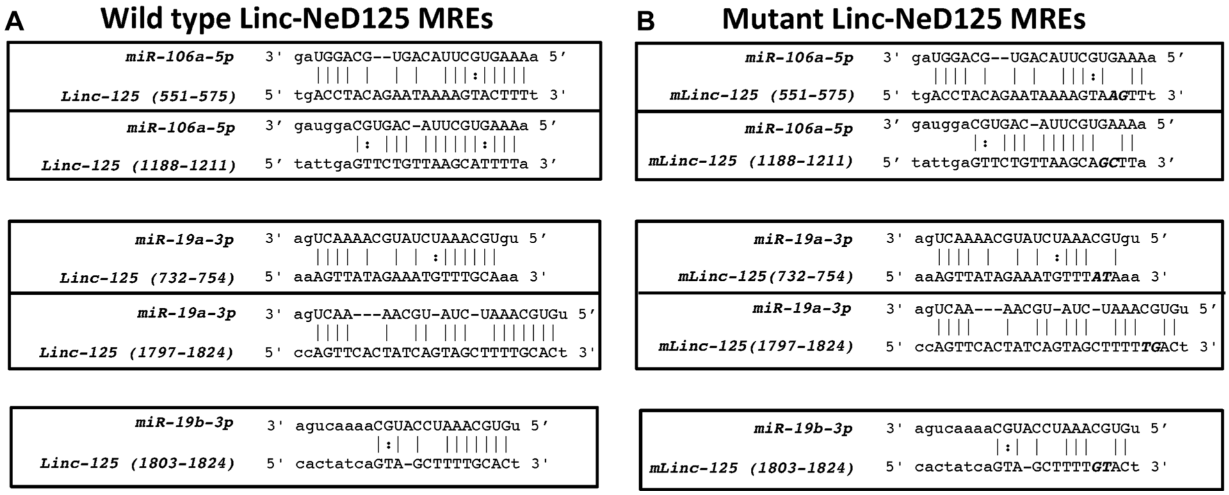

Supplementary Figure 1: Related to Figure 4A and 4B. Pairing regions between microRNAs and linc-NeD125. Lines and dots indicate Watson-Crick or non-canonical pairings, respectively. Point mutations are shown in bold/italics.

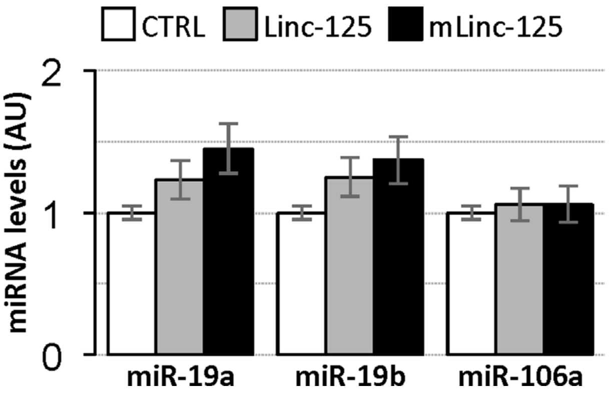

Supplementary Figure 2: Related to Figure 4B. Analysis of miRNA levels upon lincNeD-125 ectopic expression in D283 Med cells. miR-19a-3p, miR-19b-3p, miR-106a-5p levels measured in D283 Med cells transfected with wild type (Linc-125, gray bar) or mutant (mLinc-125, black bar) linc-NeD125 or empty vector (CTRL, white bar). Results (means+/-s.d.) expressed in arbitrary units (AU) are normalized vs the expression levels of U6 snRNA and referred to CTRL samples, set as 1.

Supplementary Table 1: List of all predicted microRNA binding sites along linc-Ned125, according to miRanda 3.3. See Supplementary_Table_1

Supplementary Table 2: List of human MB cases with clinical features analysed in this study

Patient ID	hystotype	subgroup	SEX	AGE GR	AGE
MB1	LCA	G4	M	AD	21
MB2	C	SHH	M	AD	18
MB3	C	G4	M	CH	6
MB4	LCA	SHH	F	I	2Y 7M
MB5	C	G4	M	CH	13
MB6	C	G3	M	CH	6
MB7	C	G4	M	CH	7
MB8	LCA	G3	M	I	2Y 5M
MB9	LCA	SHH	M	CH	12
MB10	D	G3	M	CH	7
MB11	D	SHH	F	CH	3
MB12	C	WNT	F	CH	5
MB13	C	G3	M	CH	6
MB14	C	G3	M	I	2
MB15	D	SHH	M	I	2
MB16	LCA	G4	M	CH	10
MB17	C	G3	F	CH	4
MB18	D	SHH	M	CH	6
MB19	LCA	SHH	M	CH	12
MB20	C	G4	M	CH	5
MB21	LCA	G3	M	CH	4
MB22	C	SHH	M	CH	4
MB23	C	G4	M	CH	11
MB24	D	SHH	F	I	2Y 7M
MB25	D	SHH	M	I	1Y 8M
MB26	D	G4	M	CH	8
MB27	A	G3	M	CH	7
MB28	C	WNT	F	CH	11
MB29	C	G4	F	CH	7
MB30	C	WNT	F	CH	11
MB31	C	G4	M	AD	17
MB32	C	WNT	F	CH	10
MB33	C	G4	M	CH	8
MB34	C	WNT	F	CH	13
MB35	C	G3	M	CH	16
MB36	C	G3	M	I	2
MB37	C	G4	F	CH	9
MB38	C	WNT	F	CH	10
MB39	C	G4	M	CH	8
MB40	LCA	G3	F	CH	3Y 7M
MB41	C	G3	F	I	2Y 3M
MB42	C	G4	M	CH	6
MB43	C	WNT	F	CH	12
MB44	C	WNT	F	I	2
MB45	LCA	G3	M	CH	5
MB46	C	G4	M	CH	8
MB47	C	G4	M	CH	13
MB48	C	G4	M	CH	9
MB49	C	G4	M	CH	10
MB50	C	G4	F	CH	15
MB51	C	G4	M	CH	15

Histotypes: C, classic; D, desmoplastic; LCA, large cell/anaplastic. Age group (AGE GR): Infant (I, < 3 y); Child (CH, 3-16 y); Adult (AD, >16 y).

Supplementary Table 3: List of all predicted binding sites on G4 driver genes, grouped and sorted by maximum number of shared sites, according to miRanda predictions (Aug. 2010 conserved/ high score dataset). See Supplementary_Table_3

Supplementary Table 4: List of specific genes for MB molecular subgroups according to Northcott et al. [21]

WNT	SHH	Group3	Group 4
WIF1	PDLIM3	IMPG2	KCNA1
TCN	EYA1	GABRA5	EOMES
GAD1	HHIP	EGFL11	KHDRBS2
DKK2	ATOH1	NRL	RBM24
EMX2	SFRP1	MAB21L2	UNC5D
		NPR3	OAS1

Supplementary Table 5: List of oligonucleotides employed in this study

Primers used for qRT-PCR and RT-PCR analyses

**Primer Name**	**Primer Sequence 5′-3′**
GAPDH FW	CACCATCTTCCAGGAGTGAG
GAPDH REV	CTTTCTCCATGGTGGTGAAGA
125 FW (up3)	CGAATGGTTGTGTTGATGTTCC
125 REV (pe2)	TTCTTAGCAGAGGAGGTGTCTTC

Primers used for linc-NeD125 wild type constructs

**Primer Name**	**Primer Sequence 5′-3′**
UpBam	CCGGGATCCACTGAGAGCTCTCATTTTCCCC
DownNot1	ATAAGAATGCGGCCGCGTCAACAAAGTCACACTTTGTG
UpXho1	CCGCTCGAGACTGAGAGCTCTCATTTTCCCC

Primers used for linc-NeD125 mutagenesis

**Primer Name**	**Primer Sequence 5′-3′**
Mut1 (c571a_t572g)<br>AGGCTATTC	CTTGAGAGTGACCTACAGAATAAAAGTAAGTTTAAAATA<br>AAGTAGTCAG
Mut2 (g750t_c751a)	TAAAGAGTTTAAATCTAGTTAGAAAAAGTTATAGAAATG<br>TTTTAAAAGATAAGTAACAGATAGAGTCAG
Mut3 (t1207g_t1208c)	TAATTGGTATTTATTGAGTTCTGTTAAGCAGCTTACATA<br>TTAACTCACTTAAGCCTTTCAAC
Mut4 (g1820t_c1821g)	CTCCAGTTCACTATCAGTAGCTTTTTGACTGGTCTGCCCT

Primers used for KDM6A 3′UTR cloning

**Primer Name**	**Primer Sequence 5′-3′**
UpXho2	CCGCTCGAGTAACCCAGTTCTGCACCACT
DownNot2	ATAAGAATGCGGCCGCACTGGGCACTGTACATAAAGTC

Primers used for CDK6A 3′UTR cloning

**Primer Name**	**Primer Sequence 5′-3′**
UpXho3	CCGCTCGAGGTGGTGAACTGAAAAGGGCA
DownNot3	ATAAGAATGCGGCCGCAGTCACCTGGGGCTAAATGA

MATERIALS AND METHODS

Human tissue samples

Tissues were collected with institutional review board approval and the written informed consent of all patients or their legal representatives. All specimens were formalin-fixed, sectioned, stained with hematoxylin and eosin, and examined microscopically by pathologists for diagnosis in accordance with international consensus guidelines (Louis et al., 2014). Molecular sub-grouping of tumor samples was performed as already described (Northcott et al., 2012c). RNAs of normal human cerebella (10 samples from adults, aged 25–70 years) were from Biocat, Ambion (Applied Biosystems) and BD Biosciences.

Medulloblastoma subgrouping

As previously described, qRT-PCR amplification was performed in triplicate using TaqMan probes (Mastronuzzi et al., 2014), and the mean of the three threshold cycles was used to calculate transcript quantities (Thermo Scientific). The latter were expressed in arbitrary units as the ratio of the sample quantity to the calibrator quantity or to the mean value for control samples, as previously described (Ferretti et al., 2008). All values were normalized to four endogenous gene controls (GAPDH, ß-ACTIN, ß2-MICROGLOBULIN, and HPRT).

Cell culture

BE(2)-C cells from ATCC were cultured in RPMI medium 1640 (Gibco) supplemented with 10% fetal bovine serum, L-glutamine, and penicillin/streptomycin. Differentiation was induced by exposing cells for 4 days to 10 µM all-trans-retinoic acid (RA) (Sigma-Aldrich) (Laneve et al., 2007).

D283 Med cells from ATCC were cultured in MEM medium (Gibco) supplemented with 10% or 20% heat-inactivated fetal bovine serum, 1% sodium pyruvate, 1% non-essential amino acid solution, 1% L-glutamine, and penicillin/streptomycin.

CHLA-01 Med cells from ATCC were cultured in DMEM/F12 (GIBCO) supplemented with B27 without vitamin A (GIBCO), 20 ng/ml EGF and 20 ng/ml bFGF.

RNA extraction

Total RNA was purified using TriReagent (Applied Biosystems-Thermo Scientific) and retrotranscribed with the High Capacity cDNA Reverse Transcription kit (Applied Biosystems- Thermo Scientific).

Antibodies

The antibodies used for Western blots or CLIP assay are: anti-MLL3 (Abcam, ab71200), anti-KDM6A (Abcam, ab36938), anti-SNCAIP (Atlas Antibodies HPA003266), anti-NMYC (Santacruz, C-19) anti-CDK6 (Santacruz, SC-7961), anti-GAPDH (Abcam, ab8245), anti-AGO2 (Ascenion, 11A9), anti-KI-67 (Abcam, ab16667). HRP-conjugated secondary antibodies (Santa Cruz Biotechnology) were used in combination with enhanced chemiluminescence (ECL Amersham).

High-throughput microRNA analysis

cDNA synthesis was carried out with the TaqMan® MicroRNA Reverse Transcription Kit (Applied Biosystems) by using equivalent fractions of RNAs from two independent pull-down assays. Amplification signal detection was carried out using the Applied Biosystems 7900HT Fast Real-Time PCR System. The values obtained were normalized versus the levels of ath-miR159a (MI000338), used as a spike-in. Two independent high-throughput qRT-PCR experiments were carried out and replicates were omitted when undetermined (Ct≥40).

Proliferation, migration and invasion assays

For proliferation assays, cells were transfected for 48hrs, and BrdU (Roche) was added to the culture medium during the last 14hrs. Cells were then plated on poly-lysine-coated chamber slides (Lab-Tek) for 3hrs and fixed with 4% PFA. BrdU positive cells were stained according to manifacturer's instructions and nuclei were counterstained with the Hoechst (H6024 Sigma). Cells were counted in triplicate and the number of BrdU-positive nuclei was annotated.

For both migration and invasion assays, D283 Med cells were transfected for 24 h as indicated in Materials and Methods. For migration assays 2.5×10^5 cells were plated in the top chamber (Boyden) with a non-coated membrane (8 µm pore size, BD Bioscences, CA, USA). For invasion assays 2.5×10^5 cells were plated in the top chamber with Matrigel-coated (Growth Factor Basement Membrane Matrix, Corning) membrane. In both assays, cells were plated in medium without serum, and medium supplemented with 20% serum was used as a chemo-attractant in the lower chamber. The cells were incubated for 24 hrs and cells that did not migrate or invade through the pores were removed by a cotton swab. Cells on the lower surface of the membrane were fixed and stained with Hoechst and counted in a Zeiss Axioscope microscope (Zeiss). The number of migrating or invading cells from three/four independent experiments were counted in 5 view-fields per membrane and the values averaged.

Bioinformatics analysis

MiRNA binding sites on linc-NeD125 were predicted with miRanda 3.3 with default parameters (John et al., 2004). MiRNA mature sequences were downloaded from miRBase 21. MiRNA/target predictions were performed with microRNA.org database (August 2010 release).

Linc-NeD125 mutant derivative (mLinc-125) carrying dinucleotide transversions in the miR-19a-3p, miR-19b-3p and miR-106a-5p binding sites was designed, avoiding mutations that formed new miRNA-binding sites, according to miRanda 3.3 predictions to the latest miRBase mature miRNA dataset.

REFERENCES

1. Ferretti E, De Smaele E, Miele E, Laneve P, Po A, Pelloni M, Paganelli A, Di Marcotullio L, Caffarelli, E, Screpanti, I, et al. Concerted microRNA control of Hedgehog signalling in cerebellar neuronal progenitor and tumour cells. EMBO J. 2008; 27:2616–2627.
2. Laneve P, Di Marcotullio L, Gioia U, Fiori ME, Ferretti E, Gulino A, Bozzoni I, Caffarelli E. The interplay between microRNAs and the neurotrophin receptor tropomyosin-related kinase C controls proliferation of human neuroblastoma cells. Proc Natl Acad Sci USA. 2007; 104:7957–7962.
3. Louis DN, Perry A, Burger P, Ellison DW, Reifenberger G, von Deimling A, Aldape K, Brat D, Collins VP, Eberhart C, et al. International Society Of Neuropathology—Haarlem consensus guidelines for nervous system tumor classification and grading. Brain Pathol. 2014; 24:429–435.
4. Mastronuzzi A, Miele E, Po A, Antonelli M, Buttarelli FR, Colafati GS, del Bufalo F, Faedda R, Spinelli GP, Carai A, et al. Large cell anaplastic medulloblastoma metastatic to the scalp: tumor and derived stem-like cells features. BMC Cancer. 2014; 14:262.
5. John B, Enright AJ, Aravin A, Tuschl T, Sander C, Marks DS. Human MicroRNA targets. PLoS Biol. 2004; 2:e363.
